# Long-Term Clinical and Imaging Findings in Patients with Lower Extremity Varicose Veins Treated with Endovenous Laser Treatment: A Follow-Up Study of up to 12 Years

**DOI:** 10.1155/2024/6829868

**Published:** 2024-02-06

**Authors:** Hossein Ghanaati, Amir Hossein Jalali, Madjid Shakiba, Diana Zarei, Nafiseh Ghavami, Kavous Firouznia

**Affiliations:** Advanced Diagnostic and Interventional Radiology Research Center (ADIR), Tehran University of Medical Science (TUMS), Tehran, Iran

## Abstract

**Introduction:**

This study investigates the long-term effectiveness and safety of endovenous laser treatment (EVLT) for chronic venous insufficiency (CVI), a condition commonly caused by dysfunctional valves in the venous circulation system.

**Materials and Methods:**

In this retrospective cohort study, patients underwent EVLT and were followed up for successive short intervals and one last time after a median duration of 9-year postprocedural. Pre- and postprocedure duplex ultrasound was used to assess changes in the great saphenous vein (GSV) diameter, reflux, and saphenofemoral junction incompetence. Quality of life was evaluated using the SF-36 and Aberdeen Varicose Vein Questionnaire (AVVQ).

**Results:**

Sixty-eight patients with a mean age of 52.4 ± 12.4 years were enrolled in the study. The mean follow-up time was 8.9 ± 2.1 years, ranging from 5 to 12 years. The mean GSV diameter significantly decreased in all patients (whole group) across proximal (from 5.8 ± 2.3 mm to 4.2 ± 2.1 mm), middle (from 4.7 ± 1.6 mm to 2.8 ± 2.2 mm), and distal (from 4.5 ± 2.3 mm to 2.2 ± 2.2 mm) segments, with *P* < 0.001. A disease recurrence rate of 33.8% was noted, predominantly in male patients and those with larger middle GSV diameters (OR = 5.2 (95%CI = 1.3-20.4) and OR = 1.5 (95%CI = 1-2.1), respectively). The average follow-up time for patients without recurrence was 8.8 ± 2.1 years. Almost half of the patients without recurrence were followed up for 10 years or more (49%).

**Conclusion:**

The efficacy of EVLT in managing varicose veins is demonstrated by its relatively low recurrence rate over a 10-year follow-up period, highlighting EVLT as a viable long-term treatment strategy.

## 1. Introduction

Chronic venous insufficiency (CVI) is a common condition caused by dysfunctional valves in the venous circulation system [1]. The lack of muscular support in the lower extremity superficial veins leads to a higher incidence of varicose veins (VVs), which is nearly 2% per year [2], higher in the lower extremities [3, 4].

VVs result from a complex pathophysiology, with saphenofemoral junction (SFJ) reflux alone responsible for 75% of cases. Additional contributing factors include venous hypertension, valvular incompetence, vein wall changes, inflammation, and shear stress alterations [4–6].

First-line management consists of the use of compression stockings, modified lifestyle, and exercise regimens [7]. Open vein surgery was previously used but had significant disadvantages [5, 7]. Currently, minimally invasive procedures like endovenous radiofrequency ablation, trans-catheter-guided sclerotherapy, and endovenous laser treatment (EVLT) are preferred [8–10]. EVLT, in particular, uses thermal energy in an outpatient setting, guided by Duplex Ultrasound, and presents fewer complications compared to traditional methods [7, 11].

Although existing research indicates that EVLT is a safe and effective treatment for VVs, there is a paucity of data on long-term outcomes concerning symptom relief, recurrence rates, and patient satisfaction. This study's objective is to examine these long-term results to evaluate EVLT's efficacy and safety more comprehensively, considering the significant impact of CVI on healthcare systems.

## 2. Materials and Methods

### 2.1. Study Design and Patient Selection

In this retrospective cohort study, 68 patients with great saphenous vein (GSV) insufficiency and reflux, who were treated by EVLT between the years of 2008 and 2016, were included. The diagnosis was made using Duplex Ultrasound, with reflux defined as the reverse flow lasting more than 0.5 seconds. In our study, Hach's classification system was employed to rate the severity of primary varicosities in the GSV. The grading scale is as follows: a score of 4 indicates varices near the groin area, 3 signifies varices around the midthigh region, 2 denotes varices in the upper calf area, 1 represents varices near the ankle, and a score of 0 is given when no varices are visible [12]. Patients were qualified for inclusion in the study if they presented with VVs accompanied by GSV reflux and were not pregnant. Exclusion criteria included age less than 18 years; patients with prior interventions on GSV; a poor health condition which was defined as the inability to perform required tasks physically, mentally, or socially; deep vein incompetency; superficial thrombophlebitis; nonhealing ulcers; nonpalpable pedal pulses; and extremely tortuous GSVs.

### 2.2. Data Collection

The radiological intervention and duplex ultrasound were conducted by expert radiologists, and data collection was undertaken by a physician. Data were collected and recorded in the patients' case report form. Patients underwent evaluations preprocedure, followed by postprocedure assessments at one week, one month, three months, and six months to discern short-term outcomes. The length of long-term follow-up, however, varied from 5 to 12 years across patients.

### 2.3. Outcomes of Study

The study focused on the diameter of the GSV, GSV reflux, the signs and symptoms, and the patients' quality of life (QoL) as variables of interest.

### 2.4. Device Description and Procedure

A preprocedural duplex ultrasound was conducted to determine the severity and tortuosity of GSV. This entails a comprehensive examination of crucial metrics like the length of time for venous reflux, the size of the veins, and the extent of vein twisting. These factors are vital because prolonged reflux duration, larger vein diameters, and heightened vein tortuosity suggest more severe venous disease. The procedure was performed using the 25 W 940 nm diode laser in continuous mode, causing thermal damage with a 1-3 mm per minute withdrawal rate.

The procedure was conducted under duplex ultrasound guidance in a conscious sedation environment. Initially, the leg was prepared and draped, followed by a targeted puncture of the GSV at the knee level, utilizing an ultrasound probe encased in a sterile cover for precision. The puncture site was carefully chosen for its proximity to the distal limit of venous reflux. Local anesthesia was administered using a 0.1–0.2% lidocaine solution provided by AstraZeneca, Wilmington, DE, USA. A 5-French (Fr) catheter was then inserted into the GSV, aided by a guidewire for exact positioning, as visualized under duplex ultrasound imaging. The catheter was advanced to a location 2 cm below the SFJ. Following this, a 600-micron laser fiber was threaded through the introducer sheath of the catheter, aiming towards the SFJ. Duplex ultrasound imaging verified the laser fiber's precise placement, supplemented by the transdermal visibility of the laser's aiming beam. The power settings for GSV less than 6, 6-8, and higher in diameter were 8, 10, and 12 W, respectively. The laser's energy damaged the vascular endothelium and caused venous obliteration.

During the laser therapy, the total energy delivered averaged 2117.6 ± 501 joules (J), with a range of 1100 to 3550 J and a median value of 2032.5 J. The duration of laser therapy varied among patients, with an average time of 184 ± 68.3 seconds (s), spanning from 84 to 595 s, and a median duration of 169 s.

### 2.5. Postprocedural Follow-Up

In this study, postprocedural management entailed a combined application of compression bandages and class 2 compression stockings exerting a pressure of 30-40 mmHg, implemented for one week postprocedure. The therapeutic effectiveness was assessed by evaluating the persistence of symptoms, occlusion of the GSV, and recanalization via duplex ultrasound. Immediate postprocedural assessments were conducted, followed by additional evaluations at intervals of one week, one month, three months, and six months. Long-term follow-ups were performed once within a timeframe of 5- to 12-year postprocedure.

Measurements of the GSV diameter were standardized across three segments: 2 cm distal to the SFJ for the proximal segment, at the midpoint between the knee and groin for the middle segment, and 2 cm proximal to the medial malleolus for the distal segment. To ensure consistency, all measurements were taken with the patient standing, during morning hours to account for circadian rhythm influences on venous diameter. The same ultrasound equipment and probe were used throughout, with measurements conducted by the same trained examiner. Additionally, calipers were positioned anteroposteriorly in each measurement to maintain uniformity in the methodology.

### 2.6. Quality-of-Life Questionnaire

Two questionnaires were used to assess the QoL in the study participants. The Medical Outcomes Study Short Form 36 Health Survey (SF-36 Iranian version questionnaire) [13] was selected to evaluate general QoL, and the Aberdeen Varicose Vein Questionnaire (AVVQ) [14] was chosen to assess disease-specific QoL in patients with VVs.

### 2.7. Statistics

Descriptive results were shown as mean ± standard deviation (median, interquartile range) or numbers and percentages as required. Variable normality was checked using the Kolmogorov-Smirnov test. Statistical tests for comparison between groups were chosen based on the normality of variables and the dependency of comparisons (parametric tests such as *t*-test, analysis of variance (ANOVA), paired *t*-test, or repeated measures ANOVA in case of normal distribution and nonparametric tests such as Mann–Whitney *U* test, Kruskal-Wallis test, Wilcoxon signed rank test, and Friedman test in case of lacking normal distribution). Comparison of nominal variables between groups was assessed by chi-square test among independent groups and Cochran's test in case of dependency. The correlation between parameters and disease recurrence was analyzed via logistic regression. IBM SPSS Statistics for Windows, version 22.0 (IBM Corp. Released 2013. IBM SPSS Statistics for Windows, version 22.0, Armonk, NY, IBM Corp.), was used for statistical analysis, with a *P* value less than 0.05 indicating statistical significance.

### 2.8. Ethics

Written consent was obtained from all patients. This study was approved by the ethics committee of a university-affiliated hospital.

## 3. Results

### 3.1. Demographic Characteristics

Sixty-eight patients with a mean age of 52.4 (±12.4) years (25-82), including 45 men (66.2%) and 23 women (33.8%), were included in this study. In our patient group, we noted an equal distribution of VVs, with 50% of the patients displaying the condition on the right side and the remaining 50% showing symptoms on the left side. In this study, it was discovered that 27 out of 68 patients (39.7%) held jobs that involved continuous standing for over one hour throughout the day.  Table 1 provides a comprehensive overview of the associated conditions and risk factors.

### 3.2. Short-Term Follow-Up

In the six-month follow-up of this research, we observed a notable decrease in the mean diameter of the GSV across all participants. The mean diameter of the proximal GSV segment reduced from 6.1 ± 2.3 mm to 3.8 ± 1.7 mm (*P* < 0.001), the middle segment dropped from 4.6 ± 1.6 mm to 3.0 ± 1.6 mm (*P* < 0.001), and the distal segment similarly decreased from 4.1 ± 2.0 mm to 2.5 ± 1.6 mm (*P* < 0.001) (Table 2).

**Figure 1:**
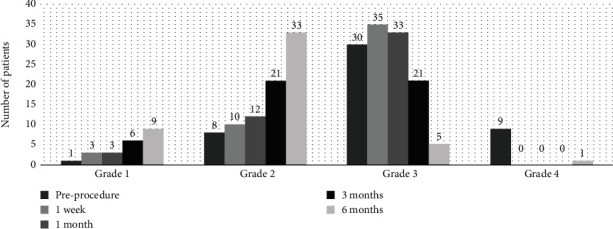
Hach grades in patients with varicose veins undergoing endovenous laser treatment in successive time sessions.

**Figure 2:**
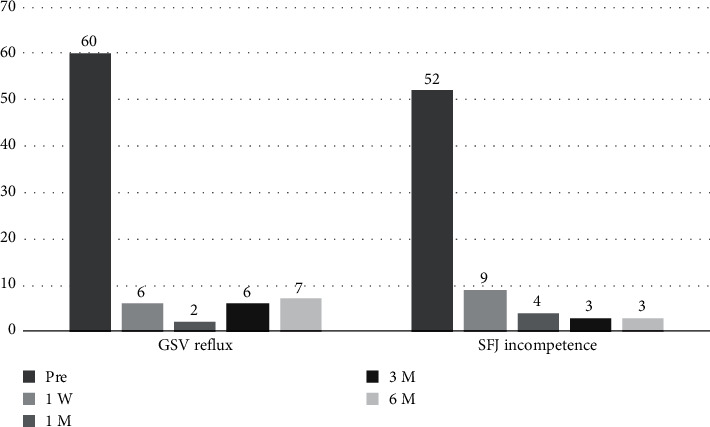
Frequency of great saphenous vein reflux and saphenofemoral junction incompetency in patients with varicose veins undergoing endovenous laser treatment in successive time sessions.

There were significant changes in the Hach classifications from before the procedure to the subsequent follow-up periods (*P* values for Hach grades comparing preprocedure to all subsequent short-term follow‐ups < 0.001). All pairwise comparisons revealed statistical significance, except the difference between the Hach classifications at the 1-week and 1-month follow-up periods (all *P* values ≤ 0.003). As illustrated in Figure 1, there is an upward trend in grades 1 and 2 over time, whereas grades 3 and 4 show a declining pattern. This indicates a decrease in Hach grades across consecutive time intervals. Among the 60 patients with data in all sessions, the number of patients showing GSV reflux dropped to 7 patients (11.7%) in 6 months' follow-up (*P* value < 0.001). In addition, a total of 52 patients showed SFJ incompetence before the procedure (86.7%), while the number of patients with SFJ incompetence decreased to 3 (5%) in 6 months' follow-up (*P* value < 0.001) (Figure 2). Furthermore, the pain measured using the numeric rating scale showed significant improvement within the first week for 87.7% of patients, and this increased to 91% after six months (*P* < 0.001).

### 3.3. Long-Term Follow Up

The mean duration of follow-up was 8.9 ± 2.1 years, varied between 5 and 12 years among patients.

Across all patients, the average diameter of the proximal segment of the GSV dropped from 5.8 ± 2.3 mm pretreatment to 4.2 ± 2.1 mm at the most recent follow-up (*P* < 0.001). Similarly, the middle GSV segment's mean diameter reduced from 4.7 ± 1.6 mm before the procedure to 2.8 ± 2.2 mm during the last follow-up (*P* < 0.001). The mean diameter for the distal GSV segment also showed a significant decline, going from 4.5 ± 2.3 mm pretreatment to 2.2 ± 2.2 mm at the final follow-up (*P* < 0.001) (Table 3).

#### 3.3.1. Quality of Life (QoL)

The results of the disease-specific questionnaire (AVVQ) demonstrated the substantial positive impact of EVLT in the long-term management of VVs. Additional and comprehensive information can be found in Supplementary Material Table 1.

The SF-36 results also demonstrate significant improvement in QoL across all domains except emotional well-being (Table 4).

#### 3.3.2. Recurrence Rate

Considering that recanalization and reflux are acknowledged as types of recurrence, in the latest follow-up, GSV reflux and recanalization were seen in 23 out of 68 patients (33.8%). A more detailed examination of the data reveals that the minimum incidence of GSV reflux occurred one-month postprocedure, with only 3 reported cases. Among the 23 patients who developed GSV reflux, 7 instances occurred within six-month postintervention, while the remaining 16 cases were identified during the extended follow-up period. Our evaluation revealed no significant difference in the baseline Hach classification and proximal GSV diameter, near the groin, between patients with and without disease recurrence, with respective mean diameters of 6.1 ± 3 mm and 5.7 ± 1.8 mm (*P* = 0.5). However, the baseline middle GSV diameter was larger in the recurrence group, with a mean diameter of 5.4 ± 2 mm compared to 4.4 ± 1.3 mm in the nonrecurrence group (OR = 1.5 (95%CI = 1-2.1), *P* value = 0.03). The distal GSV diameter (near the ankle) before the procedure was also larger in the recurrence group, with a mean diameter of 5.3 ± 3 mm compared to 4.0 ± 1.7 mm in the nonrecurrence group; the difference, however, was borderline (*P* = 0.056) (Table 5). These results suggest that a larger middle GSV diameter may be a predictor of recurrence of GSV reflux after treatment. GSV reflux at the last follow-up was seen in 18 out of 41 men, accounting for 43.9% while it was seen in 3 out of 23 women (12%) (OR = 5.2 (95%CI = 1.3-20.4), *P* value = 0.012). There was no significant correlation between the mean BMI and the presence or absence of GSV reflux. Additionally, a history of DVT did not relate to the recurrence of GSV reflux in the last visit.

Totally 45 patients did not show recurrence in the last follow-up; among them, the data of follow-up time was determined in 43 patients. These 43 patients were followed up for 8.8 ± 2.1 years (range: 5-12 years). Among these 43 patients, 22 were followed up for a period of 5 to 9 years (51.2%), 15 patients were monitored for 10 years (34.8%), and 6 patients were followed up for a duration of 11 or 12 years (14%).

## 4. Discussion

EVLT is a minimally invasive and safe treatment option for patients with refractory VVs offering several benefits compared to conventional surgical treatments [15, 16]. Short-term outcomes of EVLT have been reported to be excellent, with an occlusion rate of over 90% and minimal complications [17, 18]. However, there is limited research on the long-term efficacy of laser therapy for treating VVs.

In a randomized clinical trial (RCT) study [19], patients were followed up for 5 years after treatment. The study revealed that EVLT was more effective compared to surgery.

The diameter of the GSV is a critical determinant in predicting the occurrence of reflux. According to previous research, a GSV diameter exceeding 5.05 millimeters is the strongest predictor of reflux [20]. In the current study, across all patients, we observed a notable reduction in the mean GSV diameters from the initial pretreatment measurements to the most recent follow-up. Specifically, the average diameters decreased from 5.8 ± 2.3 mm, 4.7 ± 1.6 mm, and 4.5 ± 2.3 mm in the proximal, middle, and distal segments to 4.2 ± 2.1 mm, 2.8 ± 2.2 mm, and 2.2 ± 2.2 mm, respectively.

Both EVLT and surgery are effective short-term treatments that improve the patients' QoL and symptoms. However, in the long term, patients who underwent EVLT had better clinical outcomes and higher satisfaction levels compared with surgery [21]. All patients demonstrated symptom improvement after the procedure, with significant improvements in QoL observed in various areas, except for emotional well-being, which showed a less pronounced enhancement.

According to a systematic review study conducted by Kheirelseid et.al in 2018 [22], which included all clinical trial studies that compared long-term outcomes of EVLT and surgical treatments for VVs, the recurrence rates for EVLT (36.6%) and surgical treatments (33.3%) were not significantly different, a finding that aligns with the results of the current study.

O'Donnell et al. conducted an additional systematic review [23], which examined the incidence of VV recurrence following EVLT in comparison to ligation and stripping (L&S). This analysis encompassed all RCTs with a follow-up period of at least 2 years. The study revealed that the overall recurrence rate for VVs was 22% for both EVLT and ligation and stripping (L&S), indicating a comparable rate of recurrence over time for both treatment methods.

Importantly, it should be noted that a significant majority (70%) of the patients did not experience disease recurrence. The follow-up period, averaging 9 years, was extensive, with nearly half of the patients undergoing monitoring for over a decade without recorded instances of recurrence.

Unlike previous studies [24], this study revealed a significant difference in the mean diameter of the GSV between patients who experienced a recurrence and those who did not. Specifically, patients with recurrence had a larger average diameter in the middle part of the GSV before treatment compared to nonrecurrence patients (5.4 ± 2 mm vs. 4.4 ± 1.3 mm, *P* value = 0.03).

While previous research has shown a correlation between being female and the onset of VVs [25], the present study yielded contrasting results. Specifically, a notable increase in the occurrence of VVs among men was observed, along with a higher chance of recurrence after treatment (*P* value = 0.012). This discrepancy may be due to men in our country engaging more in jobs requiring prolonged standing and physical work. Despite high BMI and a history of DVT known as VV risk factors [25], this study found no significant correlation between these factors and disease recurrence after treatment.

Although our follow-up schedule lacks regular intervals, it is essential to highlight the substantial duration of our follow-up, extending beyond 10 years for nearly half of our patients. This extended period confers a significant advantage to our study, providing valuable insights into long-term patient outcomes. In our long-term study, all patients showed a significant reduction in average GSV diameter that was concurrent and associated with improved general and disease-specific QoL scores. These outcomes demonstrate the enduring effectiveness of EVLT.

This study has several limitations that must be acknowledged. The absence of a control group treated with a classic method and the lack of randomized patient selection preclude a direct comparison with EVLT outcomes. Another constraint is the small sample size. Furthermore, the lack of a regular basis in our long-term follow-up and the variability in follow-up durations may potentially affect the robustness of the results; however, it is important to note that our patients were followed up at least 5 years, and this could be considered as a reasonable long follow-up time.

It should be taken into account that regarding our study starting date, we utilized a lower-wavelength laser for patients' treatment, a choice that might be less common nowadays. Recent devices utilize physical parameters linked to increased photocoagulation effectiveness. Advancements in technology might have prompted interventional radiologists to opt for more advanced lasers over a decade. Furthermore, as modern techniques continue to evolve, it is reasonable to anticipate that patients treated with contemporary devices may exhibit even more favorable long-term outcomes in the coming years.

Nonetheless, the pivotal focus of our study centers on the extended duration follow-up, encompassing the assessment of patient symptoms, laser effectiveness, and patients' QoL. Further research involving a larger cohort and more strictly defined follow-up periods, and the inclusion of a control group subjected to alternate treatments, is imperative to validate these results and assess the enduring effectiveness of the intervention.

## 5. Conclusion

This study highlights the potential of EVLT as a highly effective solution for long-term complications and recurrence rates associated with the treatment of VVs. This minimally invasive technique has been shown to significantly reduce patient symptoms and improve QoL.

## Figures and Tables

**Table 1:** Demographic and baseline characteristics of patients undergoing endovenous laser treatment for varicose veins.

Variables	Mean ± SD/frequency (%)
Age, mean ± SD (year)	52.4 ± 12.4 (25-82)
Gender, M/F no. (%)	45/23 (66.2/38.2)
BMI, mean (kg/m^2^)	27 ± 3.9 (20-35.5)
Previous pregnancy, no. (%)	21 (30.9)
Previous family history, no. (%)	11 (16.2%)
Smoking, no. (%)	14 (20.6)
Job status, no. (%)	
Moderate standing	38 (60.3)
Too much standing	27 (39.7)
Previous medical history, no. (%)	
DVT	10 (14.7)
Spontaneous bleeding	11 (16.2)
DM	3 (4.4)
Foot ulcer	15 (22.1)
Foot edema	55 (80.9)
Clinical manifestations, no. (%)	
Dull pain	2 (2.9)
Feeling heavy	12 (17.6)
Burning or irritating pain	5 (7.4)
Cramp	2 (2.9)
Physical exam, no. (%)	
Healed venous ulcer	28 (41.2)
Active venous ulcer	17 (25)
Skin changes	35 (51.5)
Edema	56 (82.4)

Abbreviations: SD: standard deviation; M: male; F: female; no.: number; BMI: body mass index; kg: kilograms; DVT: deep vein thrombosis; DM: diabetes mellitus.

**Table 2:** Comparison of mean great saphenous vein diameter in proximal, middle, and distal segments in varicose vein patients undergoing endovenous laser treatment: short-term follow-up results.

Location (no.)	Time session		Mean ± SD (mm) [median, IQR]	*P* value	Pairwise comparison *P* values
Proximal (55)	Preprocedure		6.1 ± 2.3 [5, 2.2]	<0.001	Preprocedure vs. 1 w = 0.036 3 m vs. 1 m = 0.002 All other comparisons < 0.001
	Short-term F/U	1 w	5.8 ± 2.5 [5, 2.5]		
		1 m	5.0 ± 2.4 [4.7, 2.5]		
		3 m	4.5 ± 2.0 [4, 2]		
		6 m	3.8 ± 1.7 [3.5, 2]		

Middle (48)	Preprocedure		4.6 ± 1.6 [4, 1]	<0.001	Preprocedure vs. 1 m = 0.014 Preprocedure vs. 3 m = 0.002 Preprocedure vs. 6 m 1 w vs. 6 m = 0.005 3 m vs. 6 m = 0.032 1 w vs. 3 m = 0.1 All other comparisons > 0.2
	Short-term F/U	1 w	4.1 ± 1.8 [4, 1.6]		
		1 m	3.5 ± 1.7 [3.5, 1.8]		
		3 m	3.4 ± 1.5 [3.5, 1.5]		
		6 m	3.0 ± 1.6 [3, 2]		

Distal (44)	Preprocedure		4.1 ± 2.0 [4, 2]	<0.001	Preprocedure vs. 3 m = 0.003 Preprocedure vs. 6 m and 1w vs. 3 m = 0.03 1w vs. 6 m = 0.001 1 m vs. 6 m = 0.049 3 m vs. 6 m = 0.1 All other comparisons > 0.3
	Short-term F/U	1 w	3.6 ± 1.8 [3.5, 2.38]		
		1 m	3.3 ± 1.9 [3, 1.9]		
		3 m	2.9 ± 1.4 [3, 2]		
		6 m	2.5 ± 1.6 [2.75, 2.5]		

Abbreviations: no.: number; SD: standard deviation; F/U: follow-up; w: week; m: month; vs.: versus; IQR: interquartile range.

**Table 3:** Comparison of mean great saphenous vein diameter in proximal, middle, and distal segments in varicose vein patients undergoing endovenous laser treatment: long-term follow-up results.

Location (no.)	Time session	Mean ± SD (mm) [median, IQR]	*P* value
Proximal (64)	Preprocedure	5.8 ± 2.3 [5, 2.5]	<0.001
	Long-term F/U	4.2 ± 2.1 [4, 2.5]	
Middle (65)	Preprocedure	4.7 ± 1.6 [4.4, 1.5]	<0.001
	Long-term F/U	2.8 ± 2.2 [2.7, 2.7]	
Distal (66)	Preprocedure	4.5 ± 2.3 [4, 2.5]	<0.001
	Long-term F/U	2.2 ± 2.2 [1.7, 3]	

Abbreviations: no.: number; SD: standard deviation; F/U: follow-up; w: week; m: month; vs.: versus; IQR: interquartile range.

**Table 4:** Comparing quality of life (QoL) changes in patients with varicose veins before and after endovenous laser treatment: insights from SF-36 questionnaire subscales.

Items	Before procedure, mean ± SD (*N* = 64) [median, IQR]	After long-term follow-up, mean ± SD (*N* = 64) [median, IQR]	*P* value
Physical functioning	70.3 ± 13.7 [75, 20]	87.8 ± 14 [90, 10]	<0.001
Role limitations due to physical health	30.9 ± 28.1 [25, 50]	81.3 ± 23.1 [87.5, 25]	<0.001
Role limitations due to emotional problems	80.2 ± 36.5 [100, 33.3]	96.9 ± 11.5 [100, 0]	0.001
Energy/fatigue	38.8 ± 8.2 [40, 15]	40.9 ± 8.7 [40, 10]	0.091
Emotional well-being	30.9 ± 7.1 [30, 8]	32.1 ± 6.9 [32, 8]	0.12
Social functioning	42.8 ± 14.1 [37.5, 12.5]	69.9 ± 13.9 [75, 12.5]	<0.001
Pain	42.0 ± 17.6 [32.5, 20]	74.6 ± 17.6 [77.5, 23.8]	<0.001
General health	45.4 ± 13.9 [45, 18.8]	51.7 ± 12.8 [52.5, 18.8]	0.009
Total physical score	188.6 ± 54.2 [183.8, 61.9]	295.4 ± 52.5 [307.5, 60]	<0.001
Total mental score	192.7 ± 45.7 [198.8, 46.1]	239.7 ± 25.3 [238.5, 29.1]	<0.001
Total QoL score	381.3 ± 81 [388.3, 105.8]	535.1 ± 67.8 [547.3, 89.8]	<0.001

Abbreviations: SD: standard deviation; QoL: quality of life; IQR: interquartile range.

**Table 5:** Comparison of preprocedure great saphenous vein diameter between patients with recurrent varicose veins and those without recurrence during the latest follow-up.

GSV segment	Patients with recurrence, mean ± SD (*N* = 23) [median, IQR]	Patients without recurrence, mean ± SD (*N* = 45) [median, IQR]	*P* value
Proximal	6.1 ± 3 mm [5, 2.3]	5.7 ± 1.8 mm [5, 2.5]	0.5
Middle	5.4 ± 2 mm [5, 2.9]	4.4 ± 1.3 mm [4, 1.5]	0.03
Distal	5.3 ± 3 mm [5, 3.7]	4.0 ± 1.7 [4, 2.3]	0.056

Abbreviations: GSV: great saphenous vein; SD: standard deviation; mm: millimeter; IQR: interquartile range.

## Data Availability

We inserted the data in the tables of the manuscript. However, if the journal asks for some other data, we can send them.

## Supplementary Materials

Supplementary materials Table 1: comparing disease-specific quality of life changes in patients with varicose veins before and after endovenous laser treatment: insights from Aberdeen Varicose Vein Questionnaire (AVVQ).
