# Supplementary material for: Long-Term Clinical and Imaging Findings in Patients with Lower Extremity Varicose Veins Treated with Endovenous Laser Treatment: A Follow-Up Study of up to 12 Years
Source: Int J Vasc Med. 2024 Feb 6;2024:6829868. doi: 10.1155/2024/6829868 (PMC10864052; doi:10.1155/2024/6829868)
Supplement: Supplementary materials — Table 1: comparing disease-specific quality of life changes in patients with varicose veins before and after endovenous laser treatment: insights from Aberdeen Varicose Vein Questionnaire (AVVQ). [file 6829868.f1.docx]

**Supplementary Material**

**Table 1. Comparing Disease-Specific Quality of Life Changes in Patients with Varicose Veins Before and After Endovenous Laser Treatment: Insights from Aberdeen Varicose Vein Questionnaire (AVVQ)**

| Questions | | Before Procedure, N. (%) | After long-term Follow-up, N. (%) | P-Value |
| --- | --- | --- | --- | --- |
| Pain or discomfort related to varicose veins | **> 3 months** | 47 (69.1) | 6 (8.8) | <0.001 |
|  | **1-3 months** | 15 9 (22.1) | 14 (20.6) |  |
|  | **Less than 1 month** | 4 (5.9) | 24 (35.3) |  |
|  | **Never** | 2 (2.9) | 24 (35.3) |  |
| Painkiller use related to varicose veins | **> 3 months** | 6 (8.8) | 0 | <0.001 |
|  | **1-3 months** | 9 (13.2) | 0 |  |
|  | **Less than 1 month** | 8 (11.8) | 12 (17.6) |  |
|  | **Never** | 45 (66.2) | 56 (82.4) |  |
| Itching | **Yes, above and below the knees** | 19 (27.9) | 4 (5.9) | <0.001 |
|  | **Yes, only below the knees** | 12 (17.6) | 7 (10.3) |  |
|  | **Yes, only above the knees** | 22 (32.4) | 9 (13.2) |  |
|  | **No** | 15 (22.1) | 48 (70.6) |  |
| Compression stocking | **Yes, every day with prescription** | 1 (1.5) | 38 (55.9) | <0.001 |
|  | **Yes, not every day with prescription** | 3 (4.4) | 21 (30.9) |  |
|  | **Yes, without prescription** | 19 (27.9) | 1 (1.5) |  |
|  | **No** | 45 (66.2) | 8 (11.8) |  |
| Any skin discoloration related to varicose veins | **Yes** | 59 (86.8) | 20 (29.4) | <0.001 |
|  | **No** | 9 (13.2) | 48 (70.6) |  |
| Any skin sensitivity or lesions on the ankle area related to varicose veins | **Yes, need for a medical consult** | 6 (8.8) | 9 (13.2) | <0.001 |
|  | **Yes, no need for a medical consult** | 20 (29.4) | 57 (83.8) |  |
|  | **No** | 42 (61.8) | 2 (2.9) |  |
| Any foot ulceration related to varicose veins | **Yes** | 24 (35.3) | 4 (5.90 | <0.001 |
|  | **No** | 44 (64.7) | 64 (94.1) |  |
| Ankle swelling related to varicose veins | **> 3 months** | 27 (39.7) | 8 (11.8) | <0.001 |
|  | **1-3 months** | 19 (27.9) | 2 (2.9) |  |
|  | **Less than 1 month** | 16 (23.5) | 19 (27.9) |  |
|  | **Never** | 6 (8.8) | 39 (57.4) |  |
| Any concern about appearance related to varicose veins | **Yes, severe concern** | 27 (39.7) | 2 (2.9) | <0.001 |
|  | **Yes, moderate concern** | 31 (45.6) | 8 (11.8) |  |
|  | **Yes, mild concern** | 9 (13.2) | 13 (19.1) |  |
|  | **No** | 1 (1.5) | 45 (66.2) |  |
| Appearances effect on choosing clothes | **Always** | 6 (8.8) | 0 | <0.001 |
|  | **Often** | 37 (54.4) | 2 (2.9) |  |
|  | **Sometimes** | 12 (17.6) | 12 (17.6) |  |
|  | **Never** | 13 (19.1) | 54 (79.4) |  |
| Any interference with work or activity (housework) | **Not able to do any of my work** | 8 (11.8) | 0 | <0.001 |
|  | **Moderate interference** | 47 (69.1) | 13 (19.1) |  |
|  | **Mild interference** | 8 (11.8) | 7 (10.3) |  |
|  | **No** | 5 (7.4) | 48 (70.6) |  |
| Any interference with leisure time, hobbies, or social life | **Not able to do any** | 5 (7.4) | 0 | <0.001 |
|  | **Moderate interference** | 51 (75) | 9 (13.2) |  |
|  | **Mild interference** | 7 (10.3) | 12 (17.6) |  |
|  | **No** | 5 (7.4) | 47 (69.1) |  |
